# A patient`s trajectory with autism spectrum disorder leading to forensic psychiatric institutionalisation

**DOI:** 10.1192/j.eurpsy.2024.1208

**Published:** 2024-08-27

**Authors:** J. A. Déri, A. Dvorak, R. Oberndorfer

**Affiliations:** ^1^Forensic Therapeutic Centre Göllersdorf, Göllersdorf; ^2^Clinical Division of Social Psychiatry, Department of Psychiatry and Psychotherapy, Medical University of Vienna, Vienna, Austria

## Abstract

**Introduction:**

Autism-spectrum-disorder (ASD) is a heterogenous neurodevelopmental condition with a wide range of symptoms. Typical deficits, such as impairment in social communication and interactions, can lead to violent behaviours. However, ASD is often underdiagnosed and little is known about patients with ASD in forensic institutions.

**Objectives:**

To highlight the diagnostic challenges and offending behaviour of people with ASD in the context of the criminal justice system (CJS) through a case report.

**Methods:**

The case report is based on exploration, third party anamnesis, medical documentations and court files

**Results:**

A 25-year-old man was placed in detention in 2021 after having committed a dangerous threat to unknown persons and was considered not guilty by reason of insanity. Since early childhood the patient presented with extreme mood swings and impulsive-aggressive outbursts that led to criminal mischief later on. During elementary school he developed concentration problems as well as specific learning deficits. Due to his deviant social behaviour, he was rejected from his peer group. Over the course of the years, he showed no significant responses to different psychopharmacological treatment approaches. His social anxiety grew and ultimately, he started experimenting with various drugs and drinking excessive amounts of alcohol, which induced multiple psychotic episodes. Due to the psychotic exacerbations, he was repeatedly admitted to psychiatric units for acute treatment, however the autistic disorder remained untreated. At the time of the crime an independent psychiatric assessor diagnosed schizophrenia simplex and multiple drug abuse. The patient had been in psychiatric treatment since the age of 5 and received multiple diagnoses such as combined personality disorder, different subtypes of schizophrenia, ADHD, Tourette syndrome, depressive disorder and ASD at the age of 14. Nevertheless, prior to his detention he had never received a complex therapy focusing on his ASD. According to the verdict he was admitted to a medium secure forensic ward in Lower Austria, where he was treated with antipsychotic and anxiolytic medication. Furthermore, he participated in the day-structuring treatment program ensuring routine.

**Conclusions:**

Neurodevelopmental disorders such as ASD often impose a diagnostic challenge, particularly without intellectual disability. This can lead to under- and misdiagnosis, inadequate treatment or even criminal behaviour. Impaired theory of mind, poor emotional regulation and problems with moral reasoning should be recognized and treated specifically early on to prevent further damage to both the individual and society.

**Disclosure of Interest:**

None Declared

